# Combined Treatment with Host-Directed and Anticytomegaloviral Kinase Inhibitors: Mechanisms, Synergisms and Drug Resistance Barriers

**DOI:** 10.3390/pharmaceutics15122680

**Published:** 2023-11-27

**Authors:** Markus Wild, Dubravka Karner, Jan Eickhoff, Sabrina Wagner, Jintawee Kicuntod, William Chang, Peter Barry, Stipan Jonjić, Tihana Lenac Roviš, Manfred Marschall

**Affiliations:** 1 Institute for Clinical and Molecular Virology, Friedrich-Alexander University of Erlangen-Nürnberg (FAU), Schlossgarten 4, 91054 Erlangen, Germany; markus.wild@uk-erlangen.de (M.W.); sabrina.wagner@uk-erlangen.de (S.W.); jintawee.kicuntod@uk-erlangen.de (J.K.); 2 Center for Proteomics, Faculty of Medicine, University of Rijeka, Brace Branchetta 20, 51000 Rijeka, Croatia; dubravka.karner@medri.uniri.hr (D.K.); stipan.jonjic@medri.uniri.hr (S.J.); tihana.lenac@medri.uniri.hr (T.L.R.); 3 Lead Discovery Center GmbH, Otto-Hahn-Str. 15, 44227 Dortmund, Germany; eickhoff@lead-discovery.de; 4 Department of Medical Microbiology and Immunology, California National Primate Research Center, University of California, 3146 Tupper Hall, 1 Shields Avenue, Davis, CA 95616, USA; wlchang@ucdavis.edu (W.C.); pabarry@ucdavis.edu (P.B.)

**Keywords:** herpesvirus infection, human cytomegalovirus (HCMV), antiviral drug development, direct-acting and host-directed antivirals, inhibitors of viral and host CDKs, combination treatment, drug synergism, mode-of-action, drug resistance

## Abstract

Despite the availability of currently approved antiviral drugs, infections with human cytomegalovirus (HCMV) still cause clinically challenging, sometimes life-threatening situations. There is an urgent need for enhanced anti-HCMV drugs that offer improved efficacy, reduced dosages and options for long-term treatment without risk of the development of viral drug resistance. Recently, we reported the pronounced anti-HCMV efficacy of pharmacological inhibitors of cyclin-dependent kinases (CDKs), in particular, the potential of utilizing drug synergies upon combination treatment with inhibitors of host CDKs and the viral CDK-like kinase pUL97 (vCDK/pUL97). Here, we expand this finding by further assessing the in vitro synergistic antiviral interaction between vCDK and CDK inhibitors towards HCMV as well as non-human cytomegaloviruses. An extension of this synergy approach was achieved in vivo by using the recombinant MCMV-UL97/mouse model, confirming the high potential of combination treatment with the clinically approved vCDK inhibitor maribavir (MBV) and the developmental CDK7 inhibitor LDC4297. Moreover, mechanistic aspects of this synergistic drug combination were illustrated on the levels of intracellular viral protein transport and viral genome replication. The analysis of viral drug resistance did not reveal resistance formation in the case of MBV + LDC4297 combination treatment. Spanning various investigational levels, these new results strongly support our concept, employing the great potential of anti-HCMV synergistic drug treatment.

## 1. Introduction

Human cytomegalovirus (HCMV) is a ubiquitous β-herpesvirus with a seroprevalence ranging from 40% to 95% in various regions of the world [1]. HCMV infection establishes a life-long latency, which typically remains asymptomatic in the immunocompetent host. Immunocompromised individuals, such as patients under stem cell or solid organ transplantation, AIDS or cancer therapy, however, often develop severe or even life-threatening symptoms [2]. Even more importantly, congenital HCMV infection (cCMV) of the immunonaïve host during pregnancy frequently results in symptomatic courses, such as severe cytomegalovirus inclusion disease and developmental defects in the neonate, including sensorineural hearing loss, mental retardation or microcephaly [3,4]. No anti-HCMV vaccine has been approved so far, although worldwide efforts towards the development of various preventive measures have been immense [5]. Currently available drugs for the treatment of HCMV infections include pharmacological inhibitors of functions of the viral DNA polymerase (i.e., ganciclovir, its oral prodrug valganciclovir, cidofovir and foscarnet), the viral terminase complex (letermovir) and, approved very recently, the viral protein kinase (maribavir). Despite the great benefit that these anti-HCMV drugs present for the management of HCMV infections, they are limited by undesirable complications, such as the development of viral drug resistance, restrictions in oral bioavailability in several cases, as well as sometimes serious side effects [6,7]. Consequently, novel therapeutic strategies against HCMV are the focus of current research efforts, including the exploration of novel targeting options, using both direct-acting antivirals (DAAs) and host-directed antivirals (HDAs) [7,8,9,10,11].

Recently, we characterized pharmacological inhibitors of cyclin-dependent kinases (CDKs) as very potent anti-HCMV investigational candidates and suggested CDK-directed targeting as a next-generation developmental strategy towards new antiviral small molecules [12,13,14,15]. In this ongoing line of research, we focused on both host CDKs, such as CDK7 (i.e., using the selective developmental inhibitor LDC4297 [16]), as well as the viral CDK ortholog pUL97 (vCDK; i.e., using the clinically approved maribavir/MBV and chemically distinct investigational pUL97 inhibitors [7,17,18]). These findings have to be seen in the context of regulatory studies highlighting the role of vCDK/pUL97 and CDKs for HCMV replication and virus–host interaction [17]. Very recently, we identified both a functional and physical interaction between vCDK/pUL97, cyclin H and CDK7, having a substantial impact on kinase activity as well as the efficiency of viral replication [19,20,21]. Importantly, our recent studies also highlighted a statistically significant synergistic antiviral effect through in vitro combination treatments, using two inhibitors directed to host CDKs and/or vCDK/pUL97 [12,13]. Drug synergy was exhibited by various chemical classes of inhibitor pairs directed to vCDK/pUL97 and CDK7 in the absence of any amplification of cytotoxicity. Also, novel examples of HCMV-specific drug synergy have been demonstrated by exclusive HDA treatments, i.e., using inhibitors against CDK2, CDK7, CDK8 and CDK9, or triple inhibitor combinations between CDKs and vCDK/pUL97 [12,13].

In this present study, we built on earlier results to substantiate this antiviral targeting concept with novel findings. Our research focused on both human and non-human CMVs, exploring examples of synergistic drug effects in both in vitro and in vivo settings. Additionally, we investigated mechanistic aspects, such as viral protein phosphorylation-dependent nuclear transport and kinase-driven viral genomic DNA replication, and moreover, we addressed the question of resistance barriers in combination treatments. The experimentation presented includes a number of methodologically distinct settings, such as reporter-based and classical antiviral analyses of antiviral compounds; the application of recombinant viruses, in particular, for in vivo mouse/murine CMV (MCMV) infection experiments; fluorescence-based confocal imaging of viral genome replication and intracellular protein shuttling; as well as an assessment of long-term drug resistance. Combined, these new data strongly confirm our previous suggestion to include CDK/vCDK inhibitors in forthcoming antiviral approaches, and we discuss the future opportunities of combination treatment concerning anti-HCMV drug development.

## 2. Materials and Methods

### 2.1. Cells and Viruses

Primary human foreskin fibroblasts (HFFs, derived as low-passage cultures directly expanded from clinical samples, Children’s Hospital, Erlangen, Germany) were maintained in Eagle’s Minimal Essential medium (MEM) supplemented with 1× GlutaMAX™ (both Thermo Fisher Scientific, Waltham, MA, USA), 10 µg/mL gentamicin and 10% fetal bovine serum (FBS, Capricorn, Ebsdorfergrund, Germany). Mouse embryonic fibroblasts (MEFs, ATCC, Manassas, VA, USA) were cultivated in Dulbecco’s modified Eagle’s medium (DMEM), supplemented with 1× GlutaMAX™, 10 µg/mL gentamicin and 10% fetal bovine serum. Guinea pig embryonic fibroblasts (GPEFs, courtesy of Mark R. Schleiss, University of Minnesota, Minneapolis, MN, USA; [22]) were grown in MEM supplemented with 1× GlutaMAX™, 10 µg/mL gentamicin and 10% fetal bovine serum. NIH3T3 (ATCC, Manassas, VA, USA) cells were grown in DMEM supplemented with 1× GlutaMAX™, 10 µg/mL gentamicin and 10% fetal bovine serum. All types of cultured cells were maintained at 37 °C, 5% CO_2_ and 80% humidity, and were regularly monitored for the absence of mycoplasma contamination (Lonza™ Mycoalert™, Thermo Fisher Scientific, Waltham, MA, USA). Recombinant cytomegaloviruses, i.e., HCMV strain AD169 (variant UK), expressing green fluorescent protein (AD169-GFP, [23]), murine MCMV-UL97, in which the coding region for MCMV kinase pM97 (ORF-M97) was replaced by HCMV ORF-UL97; [24], guinea pig CMV expressing GFP (GPCMV-GFP; [22]) and rhesus macaque CMV expressing GFP (RhCMV-EGFP; [25]), were used for in vitro replication assays. In vivo experiments in the MCMV/mouse model were performed with MCMV-UL97.

### 2.2. Antiviral Compounds

Antiviral drugs were obtained from the following sources: Gö6976 (Calbiochem, Darmstadt, Germany); LDC4297 (Lead Discovery Center GmbH, Dortmund, Germany); ganciclovir, maribavir (MedChemExpress, Monmouth Junction, NJ, USA); and Ax7396, Vi7392 (Vichem Kft, Budapest, Hungary). Stock aliquots were prepared in sterile DMSO (Sigma Aldrich, St. Louis, MO, USA) and stored at −20 °C.

### 2.3. Animal Experimentation

Female Balb/cAnNCrl mice were purchased from Charles River Laboratories (Wilmington, MA, USA) or bred at the laboratory mouse facility of the faculty of medicine, University of Rijeka, Rijeka, Croatia. Mice were maintained under specific pathogen-free conditions and utilized between 5 and 9 weeks of age. Caging was performed in groups of six mice maximum and body weight was monitored daily. Animals were infected with MCMV-UL97 [24] at 1.0 × 10^8^ PFU intraperitoneally (i.p.) in a final volume of 100 µL PBS or remained mock-infected at d 0. Antiviral compounds were administered daily (d 0 to d 3), either via oral gavage using feeding needles (p.o.) or via the intraperitoneal route (i.p). A solution of 20% PHOSAL^®^ 50 PG (Lipoid GmbH, Ludwigshafen, Germany) in PBS was used as solvent and vehicle control for the p.o.-treated cohort; a solution of 2.5% DMSO + 5% transcutol (Gattefossé, Saint-Priest Cedex, France) in 0.9% NaCl in PBS was used as solvent and vehicle control for the i.p.-treated cohort. Mice were sacrificed at 4 d p.i., and spleen, heart, liver and lung were dissected, subdivided into experimental bits and stored at −80 °C. Experimental protocols were reviewed and approved by the Regierung von Unterfranken, Würzburg, Germany (permit 55.2-2532-2-416; 2017–2022).

### 2.4. Mouse Organ Homogenization and Plaque Reduction Assay on MEFs

For performing plaque reduction assays, frozen spleen, heart and lung tissues were used for preparation by homogenizing in 1 mL DMEM using a Precellys 24 homogenizer (Bertin Technologies, Montigny le Bretonneux, France). MEF cells were seeded at 1.5 × 10^5^ cells/well in 12-well cell culture plates and infected on the following day utilizing dilutions of organ homogenates. After 90 min of adsorption, DMEM supplemented with 0.3% agarose (Serva, Heidelberg, Germany) was added. Agarose was removed 5–7 d p.i., depending on plaque formation. Cell layer was fixed and stained with 1% crystal violet (Serva, Heidelberg, Germany) in 20% ethanol solution, followed by three washing steps using PBS, drying and counting of plaques with an inverted light microscope.

### 2.5. Drug Interaction Assessment via Loewe Additivity Fixed-Dose Assay Adapted to HCMV-GFP In Vitro Infection

Loewe Additivity Fixed-Dose Assay was performed using an adapted protocol of the HCMV GFP-based replication system [16,23] described previously [13]. Briefly, HFFs were seeded at 1.5 × 10^5^ cells/well in 12-well cell culture plates (four plates per assay) and infected on the following day with HCMV AD169-GFP [23] in a dilution resulting in 25% GFP-positive cells at 7 d p.i. (i.e., 1× TCID_25_ 7d). After virus adsorption, the inoculum was replaced by medium supplemented with single compound, compound combination or solvent control. All infections were performed in biological duplicates. Cells were lysed by the addition of 200 µL lysis buffer/well 7 d p.i., and cell suspensions were mixed and transferred to a 96-well plate. Centrifugation was performed at 3000 rpm for 15 min and clear lysates were subjected to automated GFP quantitation in a Victor X4 microplate reader (PerkinElmer, Waltham, MA, USA). Antiviral efficacy (mean of duplicate measurement of biological duplicates) was expressed as the percentage of solvent control and entered into the CompuSyn software (Version 1.0 [26]; ComboSyn, Inc., Paramus, NJ, USA). Only experiments with an r value > 0.90 and EC_50_ values close to previously determined concentrations were accepted.

### 2.6. Drug Interaction Assessment via Loewe Additivity Fixed-Dose Assay Adapted to Non-Human GFP-Expressing Reporter CMVs In Vitro Infection

Drug interaction in the GPCMV and RhCMV in an in vitro setting was assessed using an identical protocol as described previously for HCMV (2.5). GPEF cells were infected with GPCMV-GFP, HFFs were infected with RhCMV-GFP, cells were lysed at d7 and GFP fluorescence was measured as a correlate of viral replication.

### 2.7. Drug Interaction Assessment via Loewe Additivity Fixed-Dose Assay Adapted to Plaque Reduction Read-Out of Non-Human CMVs

Drug interaction in the MCMV and RhCMV in an in vitro setting was assessed using plaque reduction as read-out. For this, MEFs or HFFs were seeded at 1.5 × 10^5^ cells/well in 12-well cell culture plates (four plates per assay) and infected on the following day with MCMV-UL97 [24] or RhCMV in a dilution resulting in app. A total of 100 plaques formed at 7 d p.i. After virus adsorption, and the inocula was replaced by fresh medium supplemented with single compound, compound combinations or DMSO as a solvent control. All infections were performed in biological duplicates. At 7 d p.i., medium was removed and cells were fixed and stained by addition of 1% crystal violet (Serva, Heidelberg, Germany) in 20% ethanol solution, followed by three washing steps using PBS, drying and counting of plaques with an inverted light microscope. Antiviral efficacy (mean of duplicate measurement of biological duplicates) was expressed as the percentage of solvent control and entered into the CompuSyn software (Version 1.0 [26]; ComboSyn, Inc., Paramus, NJ, USA). Only experiments with an r value > 0.90 and EC_50_ values close to previously determined concentrations were accepted.

### 2.8. Quantitation of pUL69 Shuttling Activity via Heterokaryon Assay

To analyze the influence of inhibitors on the nucleoplasmic shuttling of viral mRNA export factor pUL69, the heterokaryon assay [27] was employed. HFFs were first infected with HCMV-AD169 at MOI of app. 1, and seeded 2 d p.i. at a density of 600,000 cells/well on glass cover slips in 6-well plates. At the same time, NIH3T3 cells were co-seeded at an identical density of 600,000 cells/well in the same plates. After incubation of 4 h, cells were washed once with PBS and 50% poly-ethylene glycol 8000 (Sigma Aldrich, St. Louis, MO, USA), and FKS-free DMEM was added for 2 min to induce heterokaryon formation, followed by two washing steps with PBS. Subsequently, DMEM samples containing the indicated compounds were added and incubated for 2 h, after which cells were fixed using 10% formalin in PBS (10 min, RT) and permeabilized using 0.2% Triton-X 100 (Sigma Aldrich, St. Louis, MO, USA, 20 min, 4 °C) in PBS. After four washing steps with PBS, blocking was performed via incubation with 2 mg/mL human-globulin Cohn fraction II, (Merck, Darmstadt, Germany) for 30 min at 37 °C. Primary staining was performed using Alexa Fluor 488-coupled monoclonal antibody against IE1 (Merck, Darmstadt, Germany) and polyclonal rabbit antibody against pUL69 (own repository) (60 min, 37 °C), followed by secondary staining with goat Alexa 555-coupled anti-rabbit antibody (Thermo Fisher Scientific, Waltham, MA, USA) (30 min, 37 °C). Cover slips were washed four times with PBS, mounted with VECTASHIELD^®^ mounting medium with DAPI (Vector Laboratories, Burlingame, CA, USA) and sealed using nail polish. The quantitation of pUL69 shuttling activity was performed by capturing four images per treatment condition with a TCS SP5 microscope using a 63 × HCX PL APO CS oil immersion objective lens (Leica Microsystems, Mannheim, Germany), and measuring pUL69 signal intensity in Fiji/ImageJ (version 1.52p, [28]). To this end, the DAPI image was turned to 16-bit and made binary using the ‘Threshold’ function. The directory of measurement was changed to the respective 16-bit pUL69 image using the ‘set measurement’ function, and intensity quantitated with the ‘analyze particles’ function, with ‘particle size’ set to 0.5–infinity and ‘exclude on edges’ active. Mean + SEM was calculated across all cells in the analyzed four images per treatment condition and plotted.

### 2.9. Measurement of Viral Polymerase Activity Utilizing DNA Labeling by Click Fluorescence-Conjugation

HFFs were seeded at 300,000 cells/well on cover slips in 6-well plates and cultivated until growth to confluency by 3 days. On d 3, cells were infected with HCMV-AD169 at MOI of app. 1.5. On d 4, compounds were added at the indicated concentrations, together with 10 µM 5-ethynyl-2’-deoxyuridine (EdU, Jena Bioscience, Jena, Germany) in H_2_O. After app. 36 h of incubation, cells were fixed using 10% formalin in PBS (10 min, RT) and background fluorescence was reduced with a quenching buffer (50 mM glycine, 50 mM NH_4_Cl in PBS, 10 min, RT). Cells were permeabilized using 0.2% Triton-X 100 in PBS (20 min 4 °C), and click chemistry conjugation was performed with the following setting: 1 mM CuSO_4_ (Sigma Aldrich, St. Louis, MO, USA), 5 mM Tris((1-hydroxy-propyl-1H-1,2,3-triazol-4-yl)methyl)amine (THPTA, Jena Bioscience, Jena, Germany), 7.5 mM sodium ascorbate (Sigma Aldrich, St. Louis, MO, USA), 0.5 µM AF488-Picolyl-Azide (Jena Bioscience, Jena, Germany) and incubated 2 h at RT. Subsequently, cover slips were washed three times with PBS, mounted with VECTASHIELD® mounting medium with DAPI (Vector Laboratories, Burlingame, CA, USA) and sealed using nail polish. Quantitation of newly synthesized DNA in HCMV-infected cells was performed by capturing four images per treatment condition with a TCS SP5 microscope using a 63× HCX PL APO CS oil immersion objective lens (Leica Microsystems, Mannheim, Germany), and by measuring the AF488 signal intensity in Fiji/ImageJ (version 1.52p, [28]). To this end, the DAPI image was converted to 16-bit and binary configuration using the ‘threshold’ function. The directory of measurement was changed to the respective 16-bit AF488 image using the ‘set measurement’ function, and intensity was quantitated with the ‘analyze particles’ function by selecting a setting of ‘particle size’ set to 0.5–infinity and ‘exclude on edges’ active. Mean values ± SEM were calculated across all evaluations of cells in the analyzed four images per treatment condition before these data were plotted.

## 3. Results

### 3.1. Assessment of In Vitro Synergistic Anti-HCMV Interaction between vCDK and CDK Inhibitors

**Figure 1:**
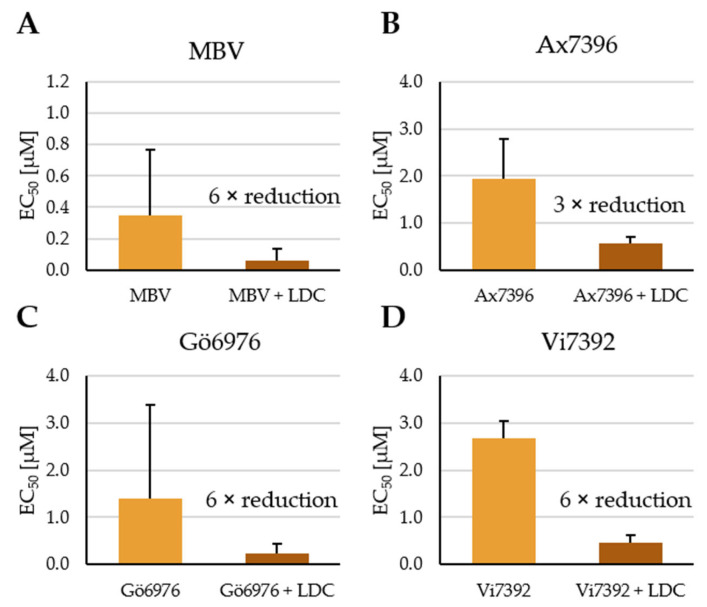
Dose reduction upon synergistic treatments using various vCDK/pUL97 inhibitors combined with CDK7 inhibitor LDC4297 (LDC), compared to single treatments with pUL97 inhibitors. (**A**) MBV (benzimodazole); (**B**) Ax7396 (quinazoline); (**C**) Gö6976 (indolocarbazole); (**D**) Vi7392 (quinazoline). HCMV AD169-GFP was used for the infection of primary human fibroblasts (HFFs) in the GFP-based replication assay, performed in a 12-well format, for the assessment of anti-HCMV in vitro efficacy of these drugs. Synergistic interactions were thus confirmed, employing the Loewe fixed-dose assay as detailed before [12]. Combinations comprised a 100:1 ratio of pUL97 inhibitor to LDC4297, and each fold reduction of the EC_50_ value of the respective vCDK/pUL97 inhibitor, which could be ascertained in the LDC4297 combination treatment, is indicated. Data are given as mean with SD over three independent experiments.

Recently, we demonstrated the pronounced antiviral activity of various CDK inhibitors [16,17,29,30,31,32,33,34,35]. Targeting HCMV specifically, combination treatment with either exclusively host-directed CDK + CDK inhibitors or both vCDK/pUL97 + CDK inhibitors displayed a statistically verified synergistic drug interaction [12,13,30]. In most analyses, primary human foreskin fibroblasts (HFFs) were used as host cells for HCMV replication because these are, compared to other cell types, more pronounced in their permissiveness for HCMV infection and have been characterized in detail [36]. Here, we focus on the synergistic antiviral efficacy of combinations between direct-acting antiviral kinase inhibitors (DAAs of vCDK/pUL97) and the host-directed antiviral LDC4297 (HDA selective for CDK7). One particular benefit of synergistic drug combinations is the potential dose reduction of the two individual drugs while maintaining high antiviral efficacy. A more detailed approach to quantitation of this in vitro dose reduction, as identified earlier [12], is illustrated here for the antiviral efficacy against HCMV (strain AD169-GFP). The effective drug combinations between the CDK7 inhibitor LDC4297 and four chemically different vCDK/pUL97 inhibitors MBV, Ax7396, Gö6976 and Vi7392 were assessed (Figure 1A–D). Half-maximal effective doses (EC_50_ values) of each of the four individual vCDK/pUL97 inhibitors alone, as well as the four combinations between vCDK/pUL97 inhibitors plus LDC4297, are given (for details of experimentation, see legend of Figure 1). As an important finding, a significant degree of drug–drug synergism (clearly distinct from the level of additive effects) could be determined for all four combinations. The weighted combination indexes (CI_wt_ values) of 0.36, 0.62, 0.42 and 0.55, respectively (Figure S1A–D; referring to Figure 1A–D), had been reported earlier [12]. Fold reduction in each EC_50_ value of the respective vCDK/pUL97 inhibitor is indicated, ranging from a six-fold to three-fold reduction of dose in combination treatment (50% inhibition of viral replication) compared to single treatment. This finding supports our earlier statement that the clinical development of vCDK/pUL97 + CDK inhibitor combination treatment possesses great potential. With LDC4297 as a suggested added cotreatment to the recently approved drug MBV, overall drug dosages might be lowered, bringing about a reduced risk of undesirable side effects.

### 3.2. Specific Synergism between the vCDK/pUL97 Inhibitor MBV and the CDK7 Inhibitor LDC4297 in Non-Human Cytomegalovirus Replication Models

The synergistic effect between LDC4297 and four chemically distinct pUL97 inhibitors indicated a true target-specific synergism based on the combined inhibition of vCDK/pUL97 and CDK7. The antiviral activity of all drugs analyzed, i.e., EC_50_ values, could be clearly differentiated from those concentration levels inducing cytotoxicity, as expressed by the respective CC_50_ and SI values [12,13]. In order to assess whether this drug interaction plays an exclusive role in HCMV replication or is similarly relevant for various CMV species, three non-human CMV replication models were used. Results for plaque reduction assays of recombinant MCMV-UL97 (see Section 3.3), as well as GFP-based replication assays for HCMV-GFP, guinea pig GPCMV-GFP and RhCMV-GFP, were collected. The calculated CI_wt_ values demonstrated a drug synergy in each of these cases, indicating that this effect is not restricted to distinct virus species or host cells (Table 1; Figure S2). MBV displayed a range of EC_50_ values between 0.348 and 36.717 µM, potentially pointing to differences in drug sensitivity of the pUL97 orthologs, especially in GPCMV and RhCMV. LDC4297-specific EC_50_ values varied to a considerably lower extent, underlining the high degree of CDK7 conservation. Weighted CI values (CI_wt_) were calculated for each combination as described elsewhere [12,13], with values above 1 indicating antagonistic, and below 1 indicating synergistic drug interaction. Consistent CI_wt_ below 1 (0.31–0.75) confirm the MBV + LDC4297 synergism in all four CMV species and thereby point to a similarly relevant regulatory interplay between vCDK/pUL97 or orthologs, and also CDK7 in non-human hosts.

### 3.3. Extension of the Synergy Approach In Vivo Using the Recombinant MCMV-UL97/Mouse Model

**Figure 2:**
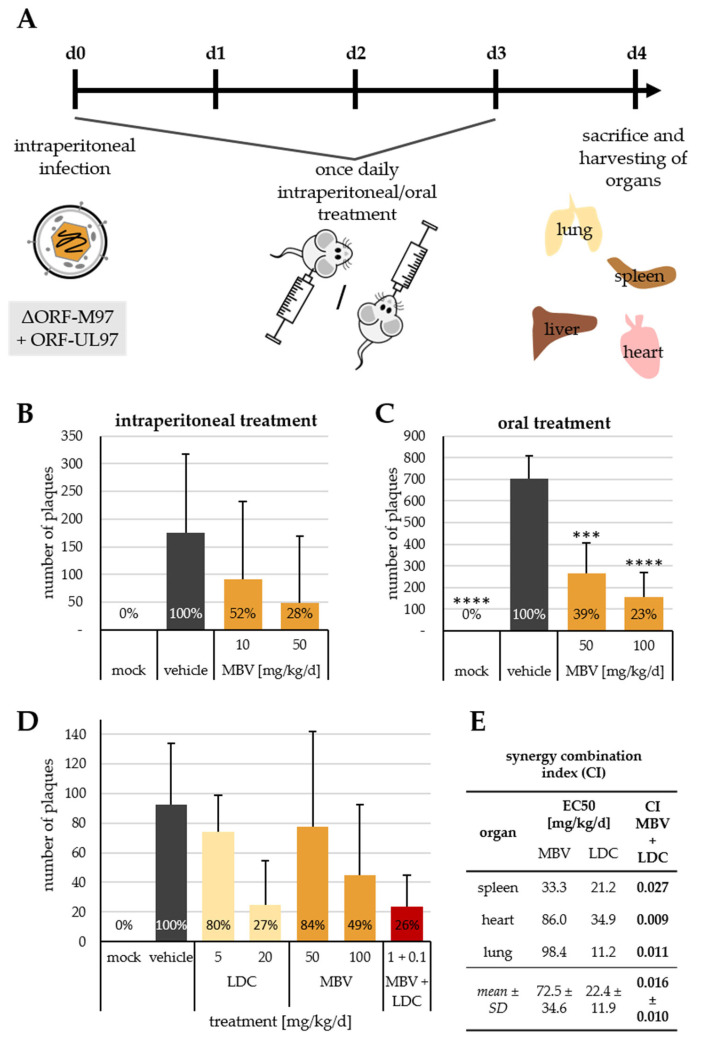
In vivo analysis of maribavir (MBV) + LDC4297 (LDC)-mediated drug synergism. (**A**) Treatment scheme for in vivo infection experiments. (**B**) MBV efficacy after intraperitoneal treatment (i.p.) using the read-out of plaque reduction assay on MEFs after infection with spleen homogenates at a dilution of 1:100. (**C**) MBV efficacy after oral treatment (p.o.) using the read-out of plaque reduction assay on MEFs after infection with spleen homogenates at a dilution of 1:100. (**D**) Efficacy of oral treatment (p.o.) with MBV, LDC and combination in vivo using the read-out of plaque reduction assay on MEFs after infection with lung homogenates at a dilution of 1:20. (**E**) Tabular summary of EC_50_ values of single treatments and CI values of combination treatments in spleen, heart and lung plaque assays. Data are given as mean with SD; statistical analysis was performed using ordinary One-way ANOVA with post hoc Tukey’s test; ****, *p* ≤ 0.0001; ***, *p* ≤ 0.001.

To translate our in vitro findings into a more complex virus–host environment of an in vivo model, the treatments with MBV and LDC4297, as well as the combination MBV + LDC4297, were investigated in mouse cytomegalovirus (MCMV)-infected Balb/c mice. Due to the high specificity in targeting HCMV pUL97, MBV is not active in inhibiting MCMV pM97 and therefore shows no antiviral effect against MCMV (Figure S3A). Consequently, the recombinant MCMV-UL97, in which ORF-M97 had been replaced by HCMV ORF-UL97, was used [24]. The efficacy of MBV against this strain was confirmed in vitro (Figure S3B) before employing the drug in the in vivo experiment. To this end, mice were infected intraperitoneally (i.p.) with the recombinant MCMV-UL97 at d 0 and treated daily from d 0 to d 4 with oral (p.o.) or intraperitoneal (i.p.) administrations (Figure 2A). The mice were sacrificed and their organs were harvested on d 4 p.i. In the setting with i.p. treatment, using 10 and 50 mg/kg/d, MBV led to a dose-dependent, albeit not statistically significant, reduction in MCMV replication to 52% and 28% of solvent control, respectively, as measured by plaque reduction assay (Figure 2B). In the parallel setting with p.o. treatment, MBV reduced infectious viral load to 39% and 23% at a concentration of 50 and 100 mg/kg/d, respectively, reaching high statistical significance (Figure 2C). These results demonstrate that the infection model utilizing the recombinant MCMV-UL97 is highly valuable in the assessment of MBV efficacy in vivo. To address the question of MBV + LDC4297 in vivo synergism, single compounds were administered at 5 and 20 mg/kg/d (LDC) and 50 and 100 mg/kg/d (MBV), and the combination MBV + LDC4297 at 1 and 0.1 mg/kg/d, respectively. Note that a low combined concentration was chosen due to the strong synergism detected in vitro and an expected comparably strong antiviral effect in vivo. A reduction of infectious viral particles was shown for all treatments using the read-out of plaque reduction assay for the assessment of organ homogenates (Figure 2D); due to high variation between the animals within groups, however, no statistical significance was achieved. Employing the Loewe additivity calculation, a synergy combination index (CI) was determined for the combination of plaque assays of the spleen, heart and lung homogenates, indicating strong synergism with values of 0.027, 0.009 and 0.011, respectively, resulting in a mean CI of 0.016 ± 0.010. This finding strongly supports our notion that MBV + LDC4297 possesses the potential to interact synergistically in an in vivo situation. Due to variations between animals and limitations of this experiment regarding group sizes and animal numbers, however, this initial proof-of-concept for MBV + LDC4297 synergy should be followed up by more extended in vivo trials.

### 3.4. Mechanistic Aspects of the Antiviral MBV + LDC4297 Synergistic Drug Activity

**Figure 3:**
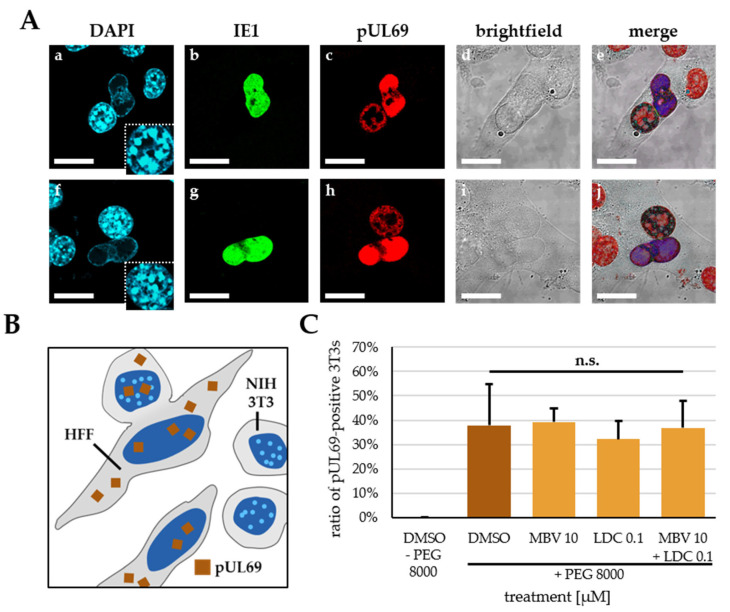
Investigation of putative influence of MBV, LDC4297 or combination treatment on nucleo-cytoplasmic shuttling of pUL69. (**A**) Exemplary images of heterokarya formed between human HFFs and mouse NIH3T3 cells, infected with HCMV AD169 and stained to visualize nuclei (DAPI) (a, f), viral proteins IE1 (b, g) and pUL69 (c, h), brightfield (d, i) and all channels merged (e, j); insets in a and f show enlarged section of NIH3T3 cell nucleus to demonstrate characteristic, punctate DNA pattern; scale bars represent 20 µm. (**B**) Schematic depiction of the heterokaryon assay to assess pUL69 shuttling activity; note one distinct heterokaryon formed between HFF and NIH3T3 cells next to two unfused NIH3T3 cells and one unfused HFF cell; pUL69 is depicted as brown squares and NIH3T3 cell nuclei are recognized by their characteristic punctate DNA pattern. (**C**) Quantitation of pUL69-positive NIH3T3 cell nuclei as measurement of pUL69 shuttling activity; data are given as mean with SD of five individual images per treatment condition, comprising at least 70 cells per treatment condition; statistical analysis was performed using ordinary One-way ANOVA with post hoc Tukey’s test; n.s., not significant.

To assess mechanistic aspects of the synergistic interaction between pUL97 and CDK7 inhibitors, two assay systems were employed. With the heterokaryon assay, the activity of the nucleo-cytoplasmic shuttling of the viral mRNA transport protein pUL69 was investigated. As pUL69 is phosphorylated by both pUL97 and CDK7 [37], it represents a candidate for the molecular link between both kinases. In this assay, infected HFFs and mouse NIH3T3 cells are densely seeded within one cocultivated layer, so that cell fusion can be investigated through the addition of fusogenic polyethylene glycol 8000 (PEG 8000). Under these conditions, heterokaryotic syncytia are produced, which contain both human and murine nuclei. Notably, the species origin of nuclei can be distinguished by DAPI staining, which characterizes murine nuclei by their punctate staining pattern, whereas human nuclei appear homogeneously stained. Thus, the characteristic DAPI staining patterns allowed for a visual identification of heterokarya carrying both human and murine types of nuclei (Figure 3A,B). In this assay, pUL69 is able to exit from human nuclei and re-enter murine nuclei within individual heterokarya due to the pUL69 nucleocytoplasmic shuttling activity. As a result of this assay, the treatment of HCMV-infected cells with DMSO, 10 µM MBV, 0.1 µM LDC4297 or a combination of MBV + LDC4297 influenced neither the frequency of heterokaryon formation (data not shown) nor the ratio of IE1-positive or pUL69-positive HFFs (Figure S4). Hence, the ratio of pUL69-positive NIH3T3 cells could be used as an indicator of pUL69 shuttling activity. In the absence of the cell fusion-causing agent PEG 8000, no NIH3T3 nucleus was found to be pUL69-positive, underlining the species specificity of HCMV (Figure 3C, first column). Treatment with 10 µM MBV, 0.1 µM LDC4297 or the combination MBV + LDC4297 in the presence of PEG 8000 showed no significant difference in the ratio of pUL69-positive NIH3T3 cell nuclei, and likewise no difference to the solvent control DMSO. An assessment of adequate protein levels achieved through the experimental conditions of HCMV infection in the protocol of pUL69-specific heterokaryon assay was performed by Western blot analysis (Figure S5; note that all expected viral proteins were detectable at appropriate levels and that under the conditions of the 2 h short-term antiviral drug treatment, no drug-mediated reduction of protein levels was produced). Thus, neither single treatment nor combination treatment with these drugs showed a measurable impact on the pUL69 nucleo-cytoplasmic shuttling activity.

**Figure 4:**
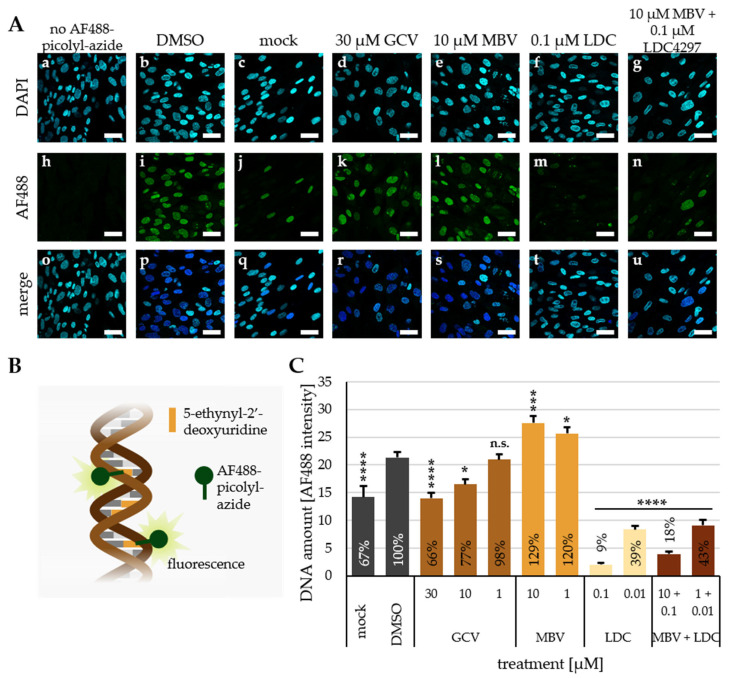
Assessment of polymerase activity via viral genomic labeling and click reaction-mediated fluorescence conjugation. (**A**) Exemplary confocal immunofluorescence images of HFFs, with newly synthesized DNA labeled with EdU and visualized with AF488 (h–n), cell nuclei (DAPI) (a–g) and merge of both channels (o–u). HFFs were seeded on glass cover slips on d 0 and used for HCMV AD169-GFP infection (MOI of 1) or remained mock-infected on d 3. Cells were treated with indicated compounds and labeled with EdU for 36 h beginning on d 4, before cells were fixed on d 6. Quenching and click reaction were performed subsequently. Scale bars represent 40 µm. (**B**) Scheme of EdU genomic labeling with click reaction-mediated conjugation of AF488-picolyl-azide. (**C**) Quantitation of labeled nuclear DNA following HCMV infection under the indicated conditions of drug treatment, as determined by genomic EdU labeling of newly synthesized DNA. The data are given as mean with SEM over at least 100 cells per treatment condition. The levels of background staining, i.e., AF488 signal in the absence of EdU labeling, were subtracted from all values. Statistical analysis was performed using ordinary One-way ANOVA, with post hoc Tukey’s test compared to DMSO. ****, *p* ≤ 0.0001; ***, *p* ≤ 0.001; *, *p* ≤ 0.05; n.s., not significant.

In the next step, a polymerase activity assay using viral genomic labeling was established. To this end, HFFs were seeded and left to grow confluent for 3 days, after which they were infected with HCMV AD169 and incubated for 36 h with compounds and 5-ethynyl-2′-2′-deoxyuridine (EdU). Due to the dense seeding and 3 d growth before infection, HFFs were completely confluent at the time of genomic labeling, and EdU was integrated mainly into newly synthesized viral genomes. After fixation, a copper-catalyzed click reaction was performed, linking the fluorescent AF488 to EdU and allowing for the quantitation of newly synthesized DNA via fluorescence (Figure 4A,B; for primary data, see a comprehensive set of captured images in Figure S8). Compared to the DMSO-treated HCMV-infected cells (DMSO, 100%), mock-infected cells showed a residual level of DNA synthesis (mock, 67%; Figure 4C), which reflects the normal activity of basic cellular DNA synthesis in the absence of HCMV. Upon HCMV infection, signal levels were found to be strongly increased (DMSO; Figure 4C), thus illustrating the massive virus-mediated onset of intracellular DNA synthesis. The reference drug GCV exhibited a dose-dependent decrease in newly synthesized DNA due to its inhibitory impact on the viral polymerase processing activity. Here, 30 µM GCV reduced the quantity of labeled DNA to 66% (a level comparable to mock-infected cells), while lower concentrations of GCV (10 or 1 µM) showed lower or no effects (77% or 98%, respectively; Figure 4C). Interestingly, MBV increased the quantity of labeled DNA to 129% and 120% at 10 and 1 µM, respectively. As the target of MBV, viral kinase pUL97 plays a crucial role in the nuclear egress of de novo-assembled viral capsids to the cytoplasm, an increase in nuclear DNA levels following MBV treatment appeared plausible under these assay conditions. LDC4297, on the other hand, led to a significant decrease in the amount of newly synthesized DNA to 9% and 39% at 0.1 and 0.01µM, respectively (Figure 4C). The combination treatment of MBV + LDC4297 showed similar results as LDC4297 alone, suggesting no synergism-specific effect on DNA synthesis (Figure 4C, columns at the right). A second, independent replicate of this experiment showed similar results, with 50 µM MBV increasing DNA amounts to 120%, and 0.5 µM LDC4297 reducing them to 42% (Figure S6; for primary data, see the captured images in Figure S9). In addition, an assessment of intracellular HCMV genome levels was also verified by HCMV-specific qPCR (for protocol, see Ref. [38]), as performed under conditions identical to the HCMV genomic labeling assay, thus basically confirming this result by applying an independent read-out system (Figure S7).

### 3.5. Absence of Viral Resistance Formation upon MBV + LDC4297 Synergistic Drug Treatment in Long-Term Settings

**Figure 5:**
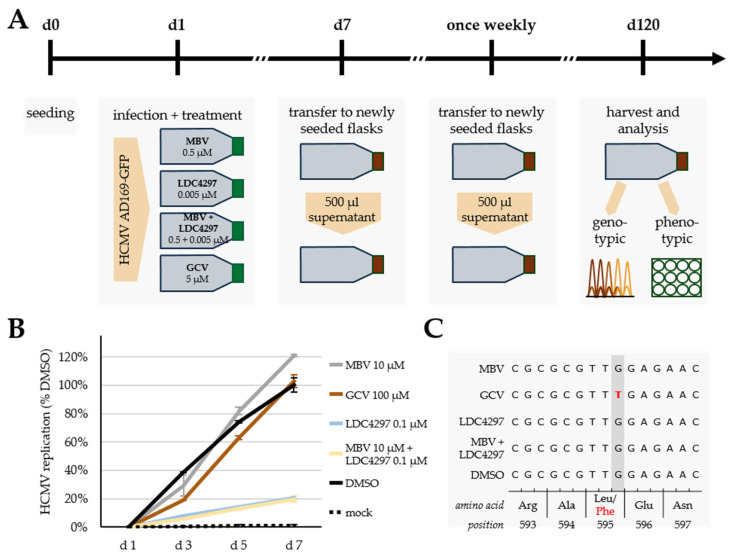
Resistance formation assessment of single compounds and combination over 120 days. (**A**) Scheme of resistance formation assay. HFFs were seeded in cell culture flasks on d 0 and infected on d 1 with HCMV AD169-GFP and treated with compounds at the indicated concentrations. Subsequently, new flasks were seeded weekly and infected with 500 µL supernatant of old flasks, renewing compound treatment at the same concentrations. On d 120, supernatants were harvested and used for further analysis. (**B**) Phenotypic analysis of resistance formation. HFFs were seeded in cell culture plates, infected using the supernatants of flasks passaged over 120 days and treated with compounds at indicated concentrations. Concentrations of compounds were chosen at levels 20-times above concentrations, under which infected cells in flasks had been passaged, in order to pose a significantly higher threshold to viral replication and to identify resistance phenotypes. GFP signal was measured on d 1, d 3, d 5 and d 7 as correlate of HCMV replication. The maximal level of solvent control DMSO was set as 100%. Data are given as mean ± SD over two biological duplicates per treatment. (**C**) Genotypic analysis of resistance formation. Viral particles were isolated from supernatant of cells infected with the supernatants of flasks passaged over 120 days; DNA was extracted from virions, and ORF-UL97 was amplified by PCR and sequenced. Genetic sequences and amino acid sequence from position 593 to 597 are depicted. A known resistance-conferring mutation (L595F) in GCV-treated sample is highlighted in red.

Formation of viral drug resistance is one of the major obstacles to success in therapeutic and preventive antiherpesviral treatment. Common experience with the occurrence of viral resistance mutations clearly points to a lower resistance barrier in the case of DAAs compared to HDAs. A combination of DAA + HDA, in particular, when acting in a synergistic fashion, might therefore significantly decrease drug resistance formation. In order to assess this point experimentally, we performed long-term HCMV infection experiments in primary fibroblast cultures, which were continuously treated with antivirals, in order to achieve a passaging of the virus under antiviral selection pressure. The experiment comprised antiviral concentrations constantly maintained at their anti-HCMV EC_50_ value (Figure 5A), the weekly transfer of infectious culture supernatants to freshly seeded cells and a harvesting of the potential yield of viral mutants by 120 d p.i. (Figure 5A). The continuous monitoring of infected cells was performed by fluorescence microscopic inspection of the viral GFP reporter in order to ensure comparable levels of infection and viral particles in the supernatant. Samples were collected and subjected to both phenotypic and genotypic resistance analyses. For the phenotypic assessment, another HCMV GFP-based antiviral assay was performed, in which the collected samples of infectious culture supernatants were used for the infection of fresh cells, again under the respective conditions of drug treatment, so that a resistant virus should be indicated by massive replication within 7 d p.i. (DMSO control was set as 100%, with infection of parental HCMV AD160-GFP). In additional control settings, the sensitivity of parental HCMV AD160-GFP to all conditions of drug treatment was monitored (for EC_50_ values and fold reduction of the drug combination, see Table 1 and Figure 1, respectively). The results revealed the formation of a GCV-resistant or MBV-resistant virus under conditions of 100 µM or 10 µM drug selection, respectively (Figure 5B), whereas the settings of LDC4297 treatment (0.1 µM) or synergistic combination of MBV + LDC4297 (10 µM + 0.1 µM) showed an unchanged drug-sensitive phenotype (Figure 5B). The genotypic analysis of sequence-specific resistance markers in the viral genome revealed a prominent GCV resistance-conferring mutation, pUL97 L595F, in the GCV group, but no comparable mutation under MBV + LDC4297 combination treatment (Figure 5C). Thus, these experiments indicated that no resistance formation was obtained with LDC4297 or with the drug combination MBV + LDC429. Contrary to our expectations, we did not detect resistance mutations under MBV monotreatment (as detected for GCV monotreatment; ORF-UL97 L595F, Figure 5C). This experimental limitation thus prevented conclusions concerning the frequency of putative drug resistance formation under combined drug treatment. Nevertheless, the absence of resistance formation in this setting of drug combination analysis supports our concept that DAA + HDA combination treatment may provide improvements in viral resistance barriers.

## 4. Discussion

In this present study, we built on our earlier finding that inhibitors of host and viral kinases, in particular, CDK7 and vCDK/pUL97 [17,32,33,34,35], provide highly promising targets for the development of a next generation of antiherpesviral drugs [16,29,30,31]. A central point of investigations was the identification of true synergistic interaction of anti-HCMV activity in the case of CDK + vCDK or CDK + CDK inhibitor combination treatment [12,13,30]. In the previous analyses, we mostly focused on providing evidence for the statistically solid basis of selected drug–drug combinations concerning their synergistic potential of anti-HCMV activity. Here, we further characterized this antiviral drug synergy in both molecular details and medically oriented aspects. Specific conclusions of this present study are as follows: (i) the antiviral in vitro synergy between CDK7 + vCDK inhibitors is detectable for human CMV and likewise for non-human, animal pathogenic CMVs; (ii) the drug synergy of CDK7 + vCDK inhibitors is also strongly supported by in vivo data using the MCMV-UL97/mouse model; (iii) mechanistic analyses of synergy point to a mode of targeting that involves the nuclear viral genomic DNA replication but not the nucleo-cytoplasmic pUL69-mediated mRNA export; and (iv) no drug resistance formation was detected for the CDK7 inhibitor LDC4297 and for MBV + LDC4297 combination treatment.

Antiviral drug combinations are routinely applied in the treatment of human immunodeficiency virus type 1 (HIV-1) and hepatitis C virus (HCV), having proven to have high cotreatment efficacy [39]. For anti-HCMV treatment, only a few studies have investigated such cotreatment options [13,40,41,42,43,44,45]. Notably, some of the studies analyzed combinations of HDAs + DAAs. As one of these examples, artemisinin derivative artemisone showed synergistic interactions with established anti-HCMV drugs (CDV, brincidofovir, MBV, GCV and LMV) [43], and in a second study, three inhibitors of cellular ribonucleotide reductase exhibited synergism when combined with GCV [44], as did LC-1310, an inhibitor of cellular ATPase p97 [13]. It should be stressed, however, that all of these studies based their results only on a single model of drug interaction and did not raise additional in vivo data for the respective antiviral combinations. Concerning our favored strategy of utilizing vCDK + CDK inhibitor synergy, as shown in this study and previous work [12,13], the investigations comprised various methodological approaches, ultimately providing refined insight into anticytomegaloviral drug combinations in vitro and in vivo, mechanistic aspects, as well as a first assessment of barriers of drug resistance formation.

As a general achievement in drug development and targeting options for antiherpesviral treatment, new paths have been opened up [5]: classically, nucleoside analogs (such as ACV, VACV, GCV, VGCV, FAM, PCV, BVD), nucleoside phosphonate analogs (CDV, BCDV) and pyrophosphate analogs (FOS) have been widely used as viral polymerase inhibitors in antiherpesviral therapies. Recently, mechanistically different targets of HCMV replication, namely viral terminase and viral protein kinase, led to the clinical approval of additional drugs (LMV, MBV) urgently needed in clinical situations [7,46]. At present, a specific focus is given to the potential of herpesviral helicase-primase inhibitors amenamevir (approved for herpes zoster treatment [47,48]) and pritelivir (in clinical phase III [49]). Additional highly interesting antiherpesviral targets have been validated on investigational and preclinical levels, with the viral nuclear egress complex (NEC) representing one of the most promising examples, as illustrated by this research group before [50,51,52,53,54,55,56,57,58,59]. A targeting option, which is directed at the strongly conserved function and structure of the herpesviral core NEC (see crystallization-based 3D structures of α-, β-, γ-herpesviral NECs in Refs. [59,60,61]), may provide an ideal opportunity for the development of a broad-spectrum antiherpesviral agent. As an extension of the numerous viral targets and DAAs taken into account for antiherpesviral drugs so far, the inclusion of host targets into the therapy strategy presents an advanced approach with the potential of resolving problems arising from viral resistance formation. So far, only a few examples of target-specific antiviral HDAs have been established in clinical treatment regimens, such as maraviroc [62], ribavirin [63] and bulevirtide [64,65], for the antiviral therapy of infections with HIV and hepatitis viruses B or D, respectively. Our concept of CDKs as novel anti-HCMV targets might broaden this current repertoire of antivirals and might include the option of HDA + DAA combination treatment. CDKs have been early recognized as important, rate-limiting host factors of the replication of herpesviruses [66,67,68], and recently, the specific importance of CDK7 for HCMV has been illustrated by reports from our group and other researchers [16,20,69,70]. In addition to CDK7, even more CDKs (in particular, CDK2, CDK4/6, CDK8) might be taken into account for further applied antiviral targeting strategies. Thus, the new results presented in this study support our statement of the great potential of combinatorial drug synergies in anti-HCMV treatment.

## 5. Conclusions

Building on our previous work, investigating CDK inhibitors as HCMV antivirals and combination treatment between DAAs and HDAs, in this study, we further characterized the synergism of simultaneous pUL97 and CDK7 inhibition. In vitro experiments using three animal pathogenic CMVs, as well as an in vivo assessment employing a recombinant MCMV, demonstrated the conserved nature of this synergistic combination. Thus, a combined antiviral therapeutic strategy might be beneficial against several herpesviruses in drug development. A long-term resistance assay showed no resistance formation for MBV + LDC4297 combination treatment, indicating options for solving current resistance problems in the application of approved and novel antiviral drugs. Mechanistic analyses employing confocal microscopy and genomic labeling revealed a significant decrease in DNA synthesis upon LDC4297 treatment, a finding that we will investigate in greater detail in the future. In summary, this study provides new insights into the synergistic pUL97 and CDK7 inhibition and confirms our strategy of DAA–HDA combinations as novel anti-HCMV treatment options.

## Figures and Tables

**Table 1:** EC_50_ value of maribavir (MBV) and LDC4297 (LDC) as well as weighted CI (CI_wt_) in HCMV, MCMV, GPCMV and RhCMV in vitro replication models.

		HCMV ^1^	MCMV-UL97 ^2^	GPCMV ^3^	RhCMV ^4^
EC_50_ [µM]	MBV	0.348 ± 0.420	1.797 ± 0.623	36.717 ± 27.270	7.532 ± 1.039
	LDC	0.009 ± 0.002	0.012 ± 0.008	0.044 ± 0.018	0.018 ± 0.017
CI_wt_		0.36 ± 0.22	0.32 ± 0.07	0.31 ± 0.01	0.75 ± 0.11

^1^ HCMV AD169-GFP-based replication assay in HFFs. ^2^ MCMV-UL97 plaque assay in MEFs. ^3^ GPCMV-GFP-based replication assay in GPEFs. ^4^ RhCMV-GFP-based replication assay in HFFs.

## Data Availability

Data are contained within the article and supplementary materials.

## Supplementary Materials

The following supporting information can be downloaded at: https://www.mdpi.com/article/10.3390/pharmaceutics15122680/s1. Figure S1. Assessment of combinations of pUL97 inhibitors MBV, Ax7396, Gö6976 and Vi7396 with LDC4297 in HCMV AD169-GFP-infected HFFs via the Loewe fixed-dose assay. Figure S2. Assessment of the MBV + LDC combination (ratio 100:1) in HCMV, MCMV, GPCMV and RhCMV in vitro replication models using the Loewe fixed-dose assay. Figure S3. In vitro assessment of maribavir (MBV) efficacy against MCMV-GFP and MCMV-UL97. Figure S4. Assessment of signal quantities referring to the HCMV pUL69-specific heterokaryon assay: ratios of IE1- and pUL69-positive HFFs. Figure S5. Assessment of protein levels referring to the HCMV pUL69-specific heterokaryon assay. Figure S6. Second experimental replicate (referring to Figure 4) in the assessment of signal quantities referring to the HCMV genomic labeling assay. Figure S7. Assessment of intracellular HCMV genome levels via qPCR, referring to the HCMV genomic labeling assay. Figure S8. Confocal images used for quantitation of genomic EdU labeling (referring to Figure 4). Figure S9: Confocal images used for quantitation of genomic EdU labeling (referring to Figure S6).
