# Supplementary material for: Combined Treatment with Host-Directed and Anticytomegaloviral Kinase Inhibitors: Mechanisms, Synergisms and Drug Resistance Barriers
Source: Pharmaceutics. 2023 Nov 27;15(12):2680. doi: 10.3390/pharmaceutics15122680 (PMC10748244; doi:10.3390/pharmaceutics15122680)
Supplement: Supplementary file 1 [file pharmaceutics-15-02680-s001.zip › pharmaceutics-2709580-supplementary.pdf]

Supplementary Materials

Combined Treatment with Host-Directed and Anticytomegaloviral Kinase Inhibitors: Mechanisms, Synergisms and Drug Resistance Barriers

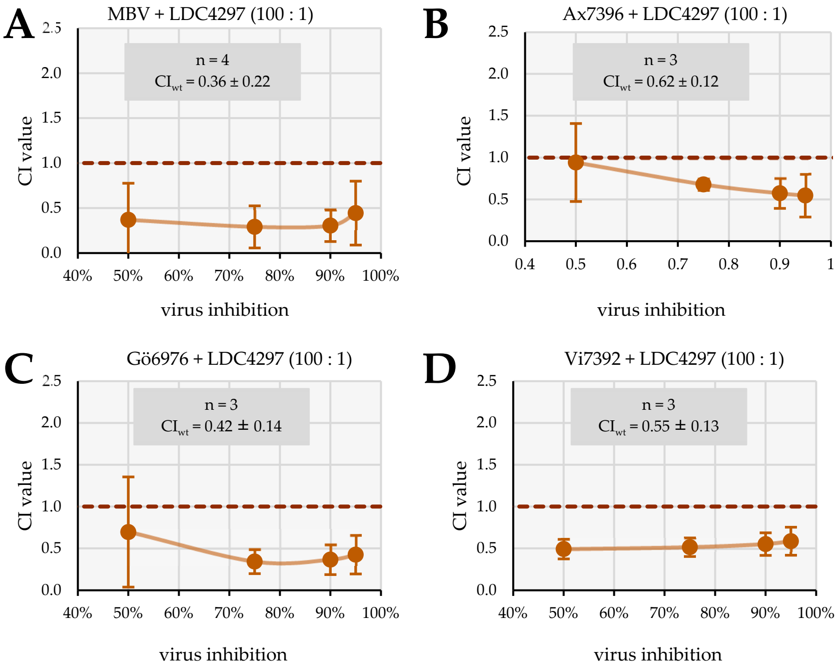

Figure S1. Assessment of combinations of pUL97 inhibitors MBV (**A**), Ax7396 (**B**), Gö6976 (**C**) and Vi7396 (**D**) with LDC4297 (ratio 100 : 1) in HCMV AD169-GFP-infected HFFs via the Loewe fixed-dose assay. Data are given as mean CI values ± SD over biological replicates at 50%, 75%, 90% and 95% virus inhibition. Number of replicates (n) and weighted CI (CI_{wt}) are given in grey boxes.

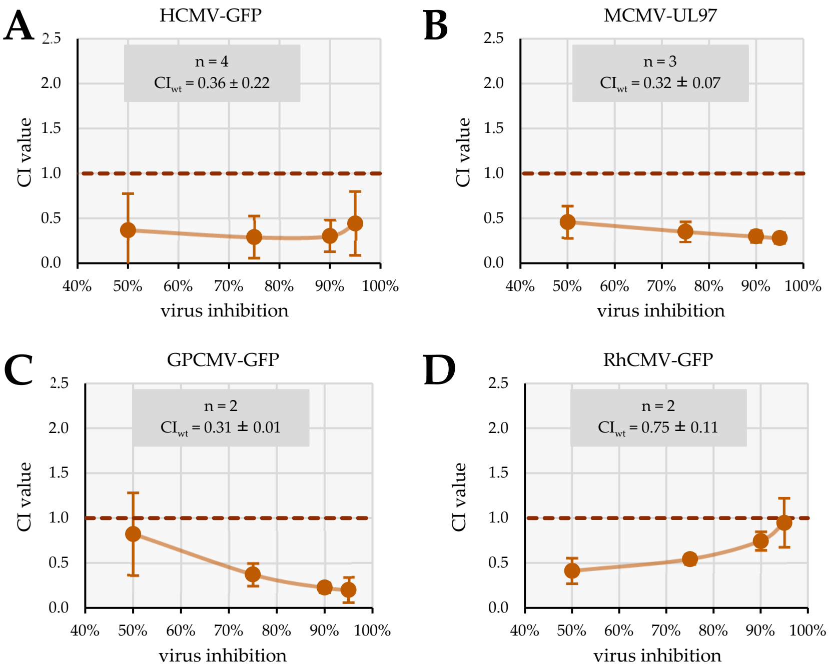

Figure S2. Assessment of the MBV + LDC combination (ratio 100 : 1) in HCMV, MCMV, GPCMV and RhCMV *in vitro* replication models using the Loewe fixed-dose assay. (**A**) HCMV GFP-based reporter assay in HFFs. (**B**) MCMV-UL97 plaque reduction assay in MEFs. (**C**) GPCMV GFP-based reporter assay in GPEFs. (**D**) RhCMV GFP-based reporter assay in HFFs. Data are given as mean CI values ± SD over biological replicates at 50%, 75%, 90% and 95% virus inhibition. Number of replicates (n) and weighted CI (CI_{wt}) are given in grey boxes.

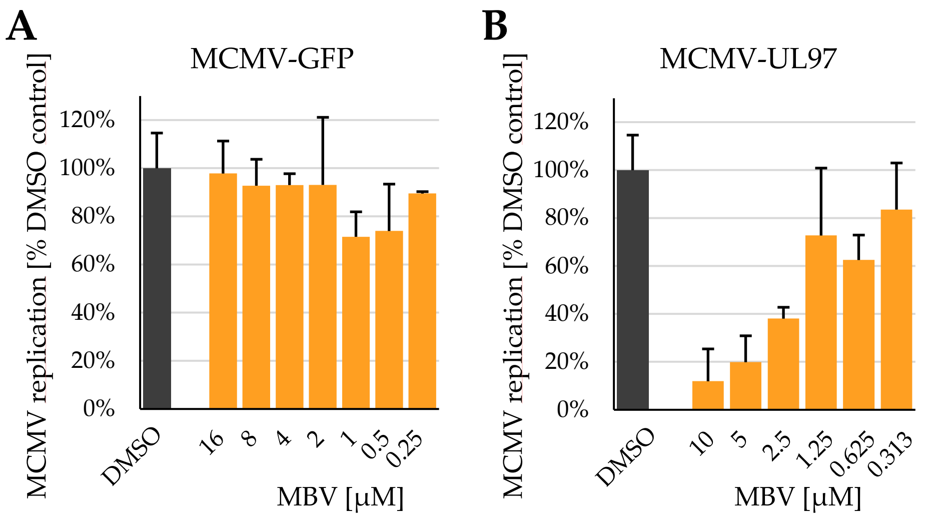

Figure S3. *In vitro* assessment of maribavir (MBV) efficacy against MCMV-GFP and MCMV-UL97. (**A**) MCMV GFP-based reporter assay in MEFs. (**B**) MCMV-UL97 plaque reduction assay in MEFs. Data are given as mean values with SD over biological duplicates.

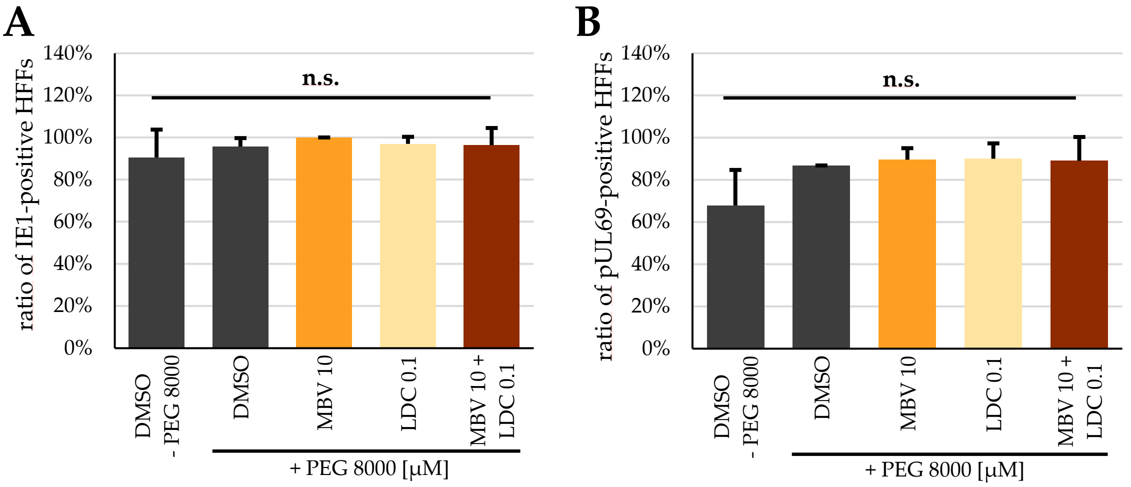

Figure S4. Assessment of signal quantities referring to the HCMV pUL69-specific heterokaryon assay: ratios of IE1- and pUL69-positive HFFs. (**A**) Ratio of IE1-positive HFFs under the applied conditions of antiviral drug treatment. Note, the lack of a significant influence of treatment on intracellular IE1 signal strength. (**B**) Ratio of pUL69-positive HFFs under the applied conditions of antiviral drug treatment. Note also here, the lack of a significant influence of treatment on intracellular pUL69 signal strength. MBV, maribavir; LDC, LCD4297.

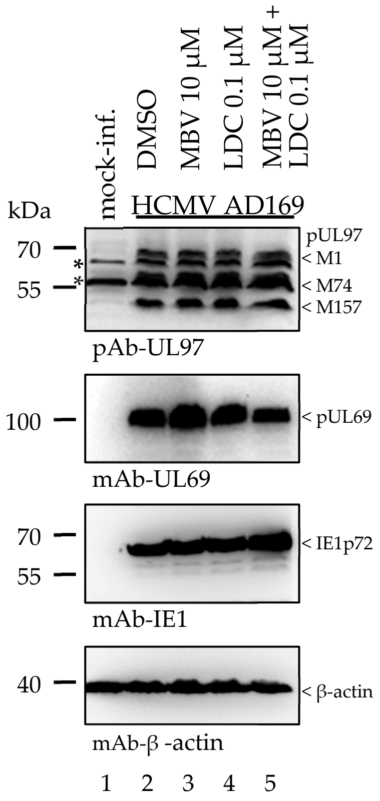

Figure S5. Assessment of protein levels referring to the HCMV pUL69-specific heterokaryon assay. Experimental setup was designed to mirror setup and time course of the heterokaryon assay, detailed in 2.8. HFFs were infected with HCMV AD169 (MOI of app. 1) or mock-infected on d 0, seeded at 2 d p.i. at 600,000 cells/well, incubated for 4 h, subsequently treated with the indicated compounds, incubated for 2 h and harvested. Cell pellets were used for SDS-PAGE and Western blot analysis. Viral proteins of interest pUL97, pUL69 and IE1p72, as well as house-keeping protein β-actin were stained using specific antibodies. pUL97 was detected in a characteristic pattern of isoforms M1, M74 and M157. MBV, maribavir; LDC, LCD4297; *, cross-reactive bands.

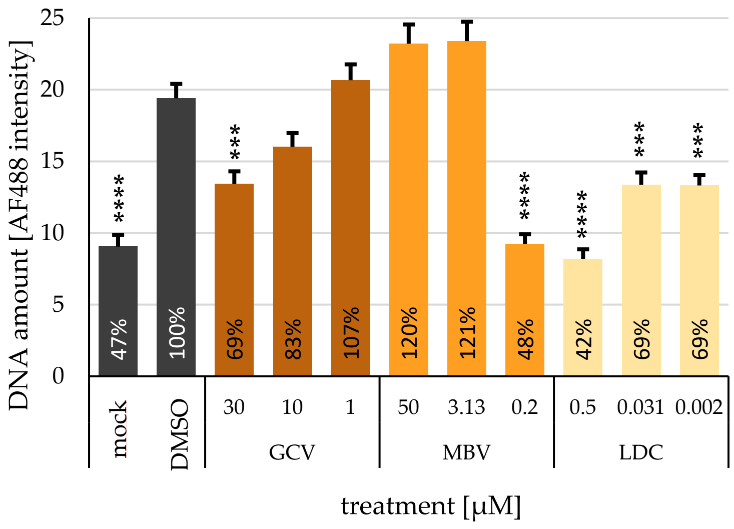

Figure S6: Second experimental replicate (referring to Fig. 4) in the assessment of signal quantities referring to the HCMV genomic labeling assay. The quantities of nuclear DNA were determined by genomic EdU labeling of newly synthesized DNA, as following HCMV infection and the applied conditions of antiviral drug treatment. Data are given as mean values with SEM over at least 100 cells per treatment condition. The levels of background, i.e. AF488 signal in the absence of EdU labeling, was subtracted from all values. Statistical analysis was performed using ordinary One-way ANOVA, with post-hoc Tukey's test compared to DMSO. ****, $p \leq 0.0001$; ***, $p \leq 0.001$; GCV, ganciclovir; MBV, maribavir; LDC, LCD4297.

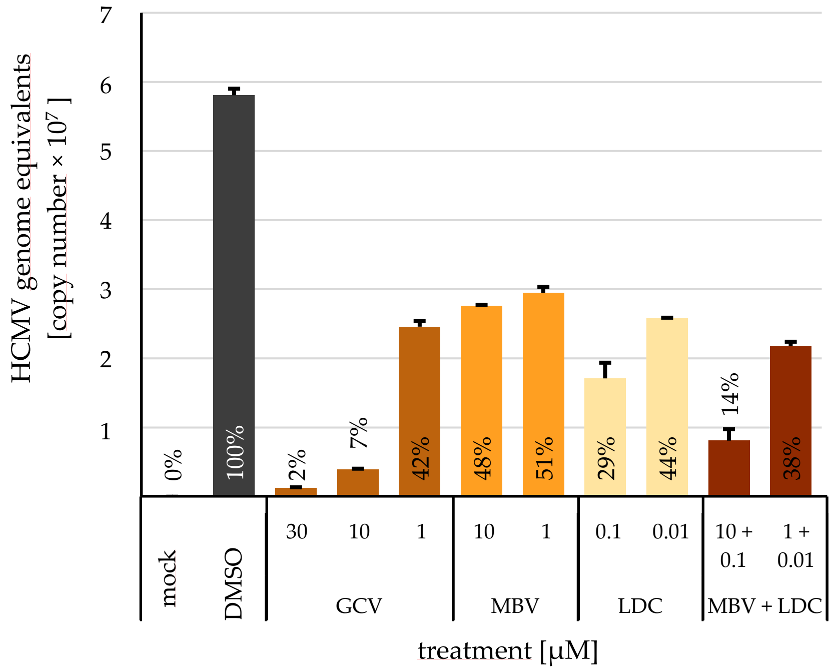

Figure S7: Assessment of intracellular HCMV genome levels via qPCR, referring to the HCMV genomic labeling assay. Experimental setup was designed to mirror setup and time course of the HCMV genomic labeling assay, detailed in 2.9. HFFs were seeded at 600,000 cells/well on d0, infected with HCMV AD169 or mock-infected on d1, treated with the indicated compounds on d2 and harvested after 36 h incubation on d3.; DNA was extracted from cell pellets using the Qiagen DNeasy Blood & Tissue kit according to manufacturer's protocol and HCMV genome equivalents were measured via specific primers and probe in a TaqMan qPCR approach. GCV, ganciclovir; MBV, maribavir; LDC, LCD4297.

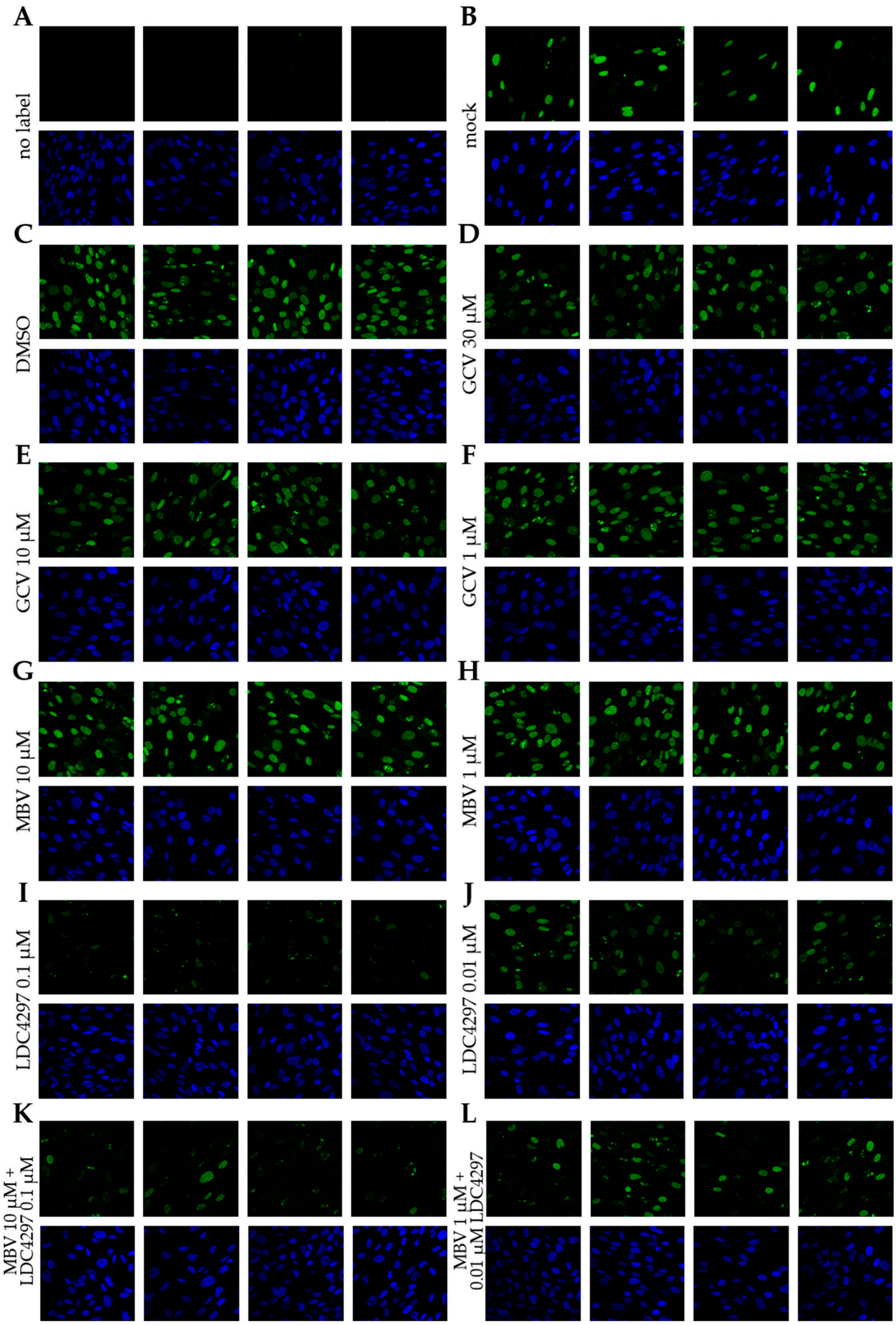

Figure S8: Confocal images used for quantitation of genomic EdU labeling (Fig. 4). Unedited images are shown for AF488 signal (green, upper levels) and DAPI signal (blue, lower levels). Experimental conditions in panels (**A**) to (**L**) are described by the labelings at the left.

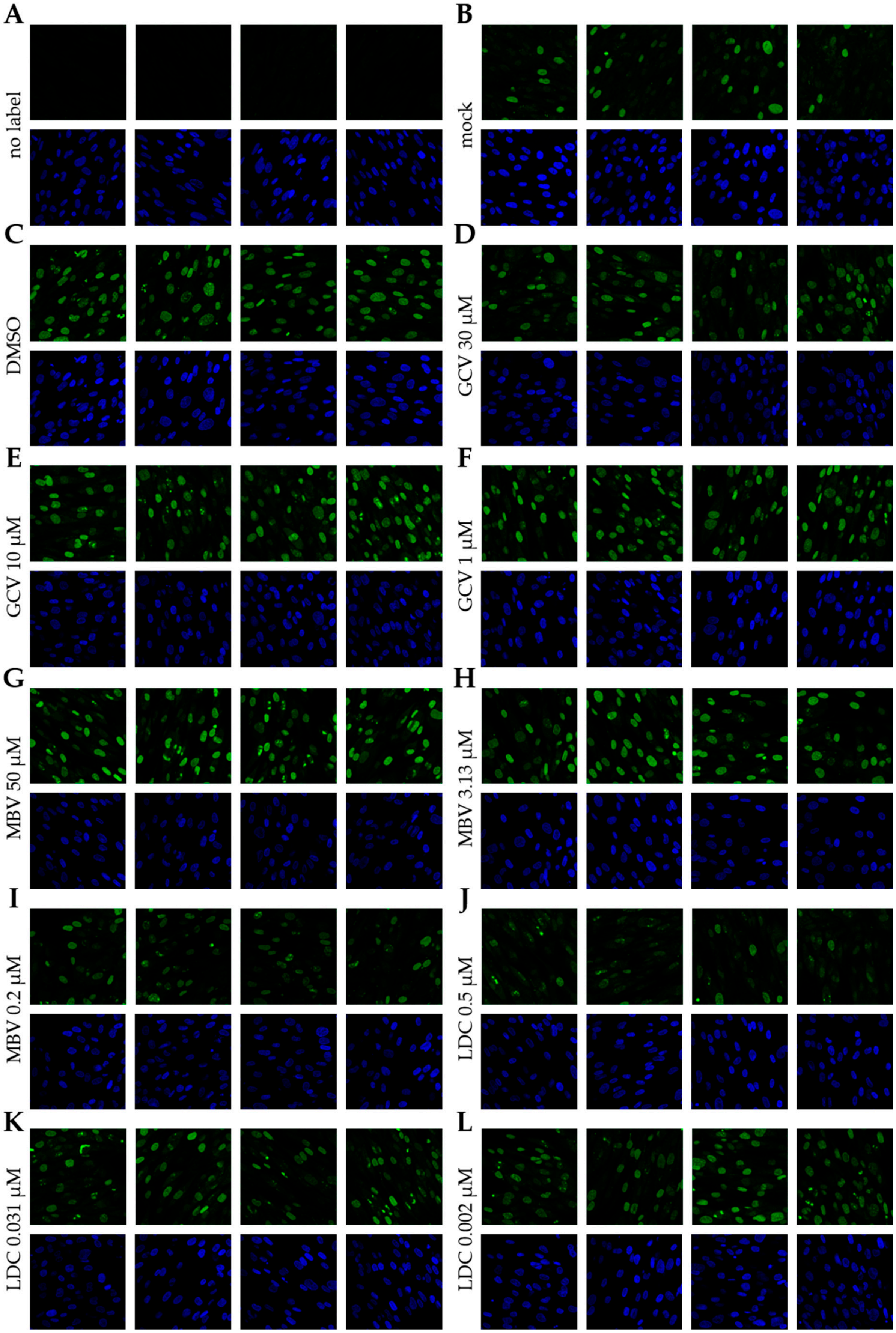

Figure S9: Confocal images used for quantitation of genomic EdU labeling (Fig. S5). Unedited images are shown for AF488 signal (green, upper panels) and DAPI signal (blue, lower panels). Experimental conditions in panels (**A**) to (**L**) are described by the labelings at the left.
